# Social determinants of health as drivers of fungal disease

**DOI:** 10.1016/j.eclinm.2023.102325

**Published:** 2023-11-18

**Authors:** Jeffrey D. Jenks, Juergen Prattes, Sebastian Wurster, Rosanne Sprute, Danila Seidel, Matteo Oliverio, Matthias Egger, Carlos Del Rio, Hatim Sati, Oliver A. Cornely, George R. Thompson, Dimitrios P. Kontoyiannis, Martin Hoenigl

**Affiliations:** a Durham County Department of Public Health, Durham, NC, United States of America; b Division of Infectious Diseases, Department of Medicine, Duke University, Durham, NC, United States of America; c Division of Infectious Diseases, ECMM Excellence Center for Medical Mycology, Department of Internal Medicine, Medical University of Graz, Graz, Austria; d BioTechMed, Graz, Austria; e Division of Internal Medicine, Department of Infectious Diseases, Infection Control and Employee Health, MD Anderson Cancer Center, University of Texas, Houston, TX, United States of America; f Faculty of Medicine and University Hospital Cologne, University of Cologne, Institute of Translational Research, Cologne Excellence Cluster on Cellular Stress Responses in Aging – Associated Diseases (CECAD), Cologne, Germany; g Faculty of Medicine and University Hospital Cologne, Department I of Internal Medicine, University of Cologne, Center of Integrated Oncology Aachen Bonn Cologne Duesseldorf (CIO ABCD) and Excellence Center of Medical Mycology (ECMM), Cologne, Germany; h German Centre for Infection Research (DZIF), Partner Site Bonn-Cologne, Cologne, Germany; i Department I of Internal Medicine, University of Cologne, Cologne, Germany; j Emory Center for AIDS Research, Emory University School of Medicine, Atlanta, GA, United States of America; k Department of Global Coordination and Partnership on Antimicrobial Resistance, World Health Organization, Geneva, Switzerland; l Faculty of Medicine and University Hospital Cologne, Clinical Trials Centre Cologne (ZKS Koln), University of Cologne, Cologne, Germany; m University of California Davis Center for Valley Fever, Sacramento, CA, United States of America; n Division of Infectious Diseases, Department of Internal Medicine, University of California Davis Medical Center, Sacramento, CA, United States of America; o Department of Medical Microbiology and Immunology, University of California Davis, Davis, CA, United States of America

**Keywords:** Social determinants of health, Fungal infections, Working conditions, Health care access, Structural conflict

## Abstract

Disparities in social determinants of health (SDOH) play a significant role in causing health inequities globally. The physical environment, including housing and workplace environment, can increase the prevalence and spread of fungal infections. A number of professions are associated with increased fungal infection risk and are associated with low pay, which may be linked to crowded and sub-optimal living conditions, exposure to fungal organisms, lack of access to quality health care, and risk for fungal infection. Those involved and displaced from areas of armed conflict have an increased risk of invasive fungal infections. Lastly, a number of fungal plant pathogens already threaten food security, which will become more problematic with global climate change. Taken together, disparities in SDOH are associated with increased risk for contracting fungal infections. More emphasis needs to be placed on systematic approaches to better understand the impact and reducing the health inequities associated with these disparities.

## Introduction

Despite efforts to improve access to quality health care, health inequities persist globally. Many factors influence disparities in health outcomes, including race, ethnic background, sex, gender identity, and sexual orientation, among others. For example, compared to white Americans, data shows that Black, Indigenous, and people of color (BIPOC) throughout the U.S. experience higher rates of morbidity and mortality from a diverse spectrum of chronic health conditions, including diabetes mellitus, hypertension, heart disease, cancer, obesity, asthma, mental health disorders, and acute conditions, such as infections.^1^^,^^2^ The ongoing coronavirus disease 2019 (COVID-19) pandemic provides a stark illustration of how underlying health inequities can be amplified by superimposed events like pandemics, leading to further increase in disease burden and health disparities.^3^^,^^4^

SDOH refer to non-medical factors that shape individuals' health outcomes and well-being. They encompass a broad range of influences and systems that impact the circumstances of people's everyday lives, including working conditions, income, housing, access to affordable and quality health services, early childhood development, education, job security, food security, social inclusion, and structural conflict.^5^ In this context, social and economic factors including education, employment, and income have a significant impact on our health behaviors and access to quality health care.^6^ These factors are further influenced and shaped by biased economic policies, development agendas, social norms, social policies, and political systems.

Globally, significant health inequities exist both within and between countries, contributing to wide gaps in life expectancy. Average life expectancy ranges from 52 years in Sierra Leone and the Central African Republic to 84 years in Japan and Hong Kong—a gap of 32 years.^7^ Even within a country, there can be striking differences in life expectancy.^8^ For instance, in Scotland, the average life expectancy for males in the less advantaged parts of Glasgow is 66 years compared to 82 years in more advantaged areas.^9^ Globally, children from the lowest income households are twice as likely to die by the age of 5 years as children born into the highest income households.^9^

Invasive fungal infections (IFIs) occur primarily in individuals with underlying immunocompromising conditions such as human immunodeficiency virus/acquired immunodeficiency syndrome (HIV/AIDS), such as those with hematologic malignancies, solid organ transplant recipients, or those critically ill in the intensive care unit (ICU),^10^, ^11^, ^12^, ^13^, ^14^, ^15^ and are overrepresented in males.^16^ While genetic and immunologic factors as well as long-term chemotherapy in individuals with cancer may play a role in the increased risk for certain IFIs in some racial and ethnic groups, such as with coccidioidomycosis in individuals with Black African or Filipino descent,^2^ it is largely believed that risk factors for IFIs are predominantly influenced by SDOH and differences in exposure to them.^17^ However, this relationship is still not fully understood.

**Fig. 1:**
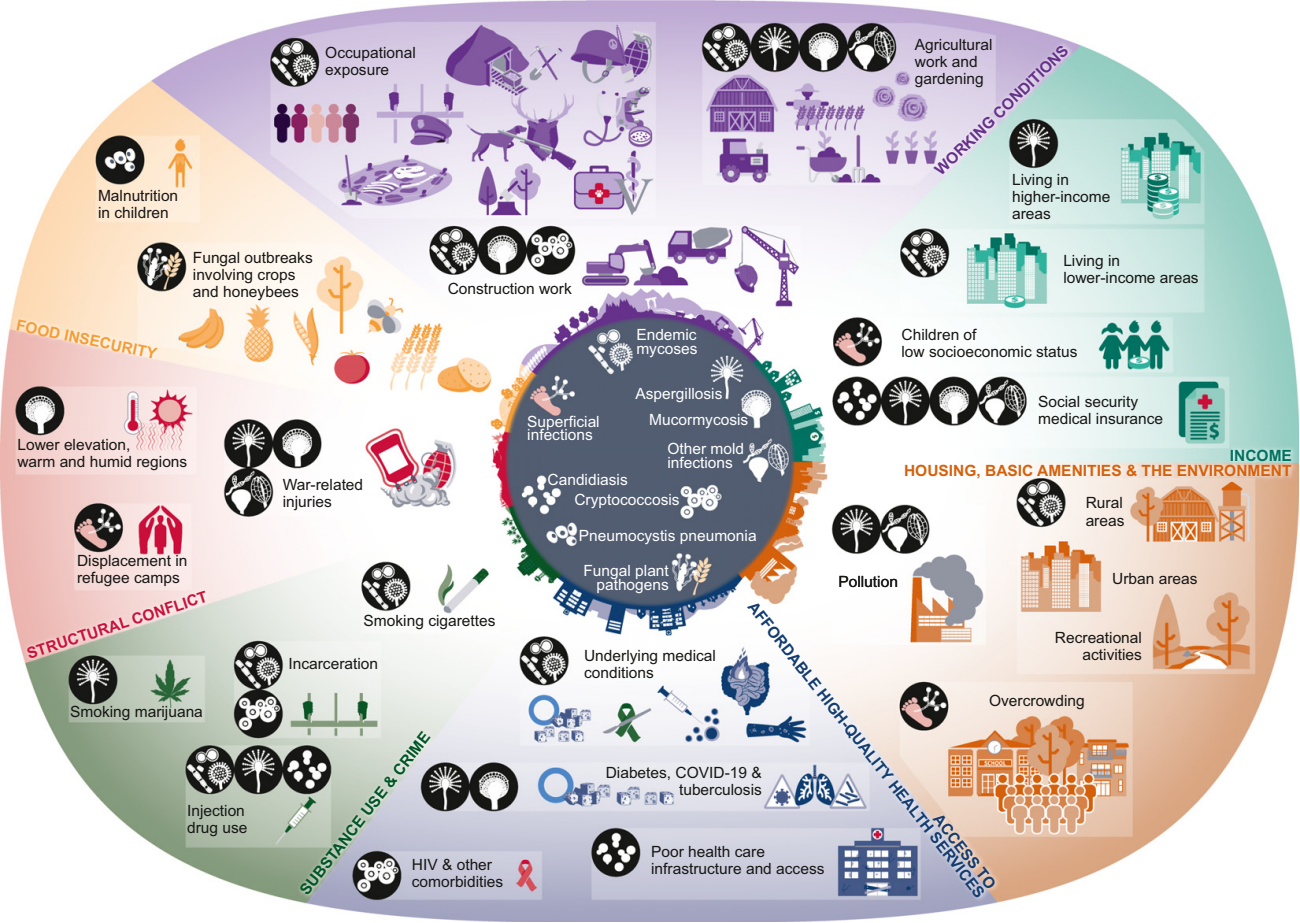
**Social determinants of health and fungal infections**.

Here, we review the literature examining how SDOH, using examples suggested by the World Health Organization (WHO)^5^ (Table 1), impact the risk for fungal infections (Fig. 1).

**Table 1:** Examples of social determinants of health as defined by the World Health Organization.^5^

Examples of social determinants of health
Income and social protection
Education
Unemployment and Job Insecurity
Working conditions
Food insecurity
Housing, basic amenities, and the environment
Substance use, crime, and incarceration
Early childhood development
Social inclusion and non-discrimination
Structural conflict
Access to affordable health services of decent quality

## Methods

### Search strategy and selection criteria

Data for this Review were identified by searches of MEDLINE, PubMed, Google Scholar, and references from relevant articles using the search terms “fungal infections”, “invasive fungal infections”, “superficial fungal infections”, “occupation”, “income”, “housing”, “healthcare access”, “incarceration”, “substance use”, “structural conflict”, and “food insecurity”. We selected relevant case reports, case series, or other studies describing cases or outbreaks of fungal infections that were related to SDOH and published in English. Searches were conducted from database inception until 14 June 2023.

### Role of the funding source

This study was unfunded.

## Social determinants of health and fungal infections

### Working conditions

Occupational factors and associated working conditions represent one of the key SDOH. Occupational exposures that increase the hazard of inhalation of fungal propagules, such as construction and agricultural work, have been shown to increase the risk for mycoses caused by dimorphic fungi in endemic regions of the U.S.^18^, ^19^, ^20^, ^21^, ^22^ Multiple studies have found an association between occupational exposure and outbreaks of coccidioidomycosis infection, including work on military bases^23^^,^^24^ or during military training exercises,^25^^,^^26^ employment in prisons,^27^ excavation at archeological sites,^28^, ^29^, ^30^ construction work,^31^, ^32^, ^33^ agricultural farm work,^34^ combating wildland fires,^18^ and work in cotton mills.^35^

Similarly, outbreaks of occupational histoplasmosis have been linked to work at an agricultural processing plant in Nebraska, U.S.,^20^ exposure to *Histoplasma* conidium in an air conditioning unit at a medical school campus,^36^ and work at a landfill and repair of a bridge in Illinois, U.S.^37^ In Alberta, Canada, an outbreak of histoplasmosis was associated with renovation of a golf course, during which soil was disturbed.^38^ A large outbreak associated with aerosol-generating tasks in caves occurred among workers in La Habana province in Cuba^39^ and among workers in the Dominican Republic who removed large amounts of bat guano from tunnels without proper respiratory protection.^40^

Occupational exposure to *Blastomyces* spp. has been reported as well. One study evaluating forestry workers in Minnesota and Wisconsin, U.S., found a high prevalence of previous subclinical infection.^21^ Other workers in endemic areas who may be at increased risk for blastomycosis include veterinarians,^41^ pathologists,^42^ or other laboratory workers.^43^ In 2023, an outbreak at a Michigan paper mill resulted in more than 100 cases of blastomycosis and at least one death.^44^ Two cases that occurred in Colorado, U.S., were associated with relocation of infected prairie dogs.^45^

*Paracoccidioides brasiliensis* can cause paracoccidioidomycosis, which most commonly occurs in countries in South America and mainly affects young and middle-aged men who work outdoors as farmers, miners, and hunters.^46^^,^^47^ One outbreak of paracoccidioidomycosis involving eight individuals in Rio de Janeiro, Brazil was thought to be due to deforestation and earth removal during construction of a highway.^22^ Infections due to *Talaromyces marneffei*, which is endemic to Southeast Asia, have also been associated with soil exposure from agricultural work and farming.^48^

Although *Aspergillus* spp. are ubiquitous in the environment and humans inhale at least several hundred *Aspergillus* conidia daily,^49^, ^50^, ^51^ high-inoculum exposures have been associated with allergic pulmonary manifestations^52^ and long-term changes to the adaptive immune environment.^53^ Occupations such as mining that involve excavation, drilling or tunneling pose a significant risk as these activities often expose workers to high concentrations of fungal propagules in the dust generated during these operations.

Occupational exposure to aflatoxin in poultry workers in Portugal has been associated with *Aspergillus* section *Flavi* contaminating indoor air in workspaces.^54^ Moreover, there have been rare cases of invasive lung infections after occupational exposures to *Aspergillus*, even in immunocompetent persons, such as from agricultural work^55^ and gardening.^56^ Cryptococcosis has been associated with outdoor occupations, such as construction and landscaping,^57^ and mucormycosis with dust exposure in a warehouse,^58^ construction^59^ and after farm work accidents.^60^ Entomophthoramycosis, which is caused by infections from fungi belonging to the order Entomophthorales, can occur following direct inoculation such as during farm work.^61^

Individuals in certain occupations may be prone to soft tissue fungal infections acquired through direct, traumatic inoculation. Sporotrichosis, caused by *Sporothrix* spp. has been associated with work in mines,^62^ forestry and tree nursery work,^63^, ^64^, ^65^ armadillo hunting in Uruguay and Brazil,^66^^,^^67^ and veterinary work.^68^ Feline sporotrichosis, caused by *Sporothrix brasiliensis*, is endemic in Brazil and neighboring countries and is a risk for veterinarians, animal caretakers, and even the general public. Infection can occur following a scratch or bite from an infected cat, although even touching an infected cat can result in infection.^69^ Infections from fungi causing mycetoma and chromoblastomycosis have been linked to farm work, agricultural work, or other outdoor manual labor.^47^^,^^70^ In endemic regions such as Sudan, fungi causing mycetoma (eumycetoma) is often found to disproportionately affect manual workers, including farmers, laborers, and herdsmen.^71^ Additionally, students in limited resource areas who frequently walk to schools without appropriate footwear or barefoot are also at higher risk.^72^ Both chromoblastomycosis and mycetoma can lead to significant disfigurement and disability which can cause considerable psychological trauma, stigma, and social isolation.^73^, ^74^, ^75^

Direct inoculation through gardening has been implicated as the cause of subcutaneous mucormycosis^76^ and mycetoma caused by *Acremonium spp.*^77^ Pythiosis, an infection caused by *Pythium insidiosum*, has been associated with agricultural work.^78^ A number of fungi causing traumatic keratitis from fungus-contaminated plant material, including *Fusarium* spp., *Aspergillus* spp., *Chrysosporium* spp., and *Curvularia* spp., has also been related to agricultural work.^79^^,^^80^

In the U.S., work that poses the greatest risk for occupational exposure to fungi employ a high percentage of BIPOC individuals, such as landscapers, groundskeepers, nursery workers, farm workers, agricultural workers, and construction workers.^81^ Notably, occupations associated with increased risk for fungal exposures and infection predominantly represent the low-wage sector^82^^,^^83^ as these workers are often unable to advocate for better working conditions or adhere to public health recommendations to prevent fungal diseases due to lack of support from supervisors. Workers who are employed in such conditions are often exposed to high rates of occupational hazards, inadequate ventilation, limited access to proper sanitation and hygiene facilities, and a lack of proper workwear and personal protective equipment (PPE).

Thus, there is a clear association between a range of fungal infections, occupations, working conditions, and the workers’ socioeconomic background.

### Income

Income, another key SDOH, is closely related to working conditions, as discussed above, as well as housing, neighborhood conditions, and access to health care. In the U.S., life expectancy has been shown to increase with income.^84^

In 2019, of 35.5 million hospitalizations in the U.S., those living in areas with the lowest quartile median income had disproportionately high hospitalization rates. IFIs overall were 1.2 times more likely to be diagnosed in individuals living in a Q1 postal code (median household income < US$48,000) compared to those in a Q4 postal code (median household income ≥ US$82,000), driven by cryptococcosis (twice as likely), and histoplasmosis (1.7 times more likely). Only aspergillosis was diagnosed more frequently in Q4 postal codes compared to Q1 postal codes, possibly related to higher rates of autoimmune diseases, hematologic disorders, and access to advanced cancer treatment and procedures such as organ transplants among hospitalized patients living in higher income areas. IFIs were also more common in hospitalizations where Medicaid was billed compared to hospitalizations where private insurance was billed.^85^

Although poorly studied on a global level, individuals belonging to lower socioeconomic groups have also been shown to be at greater risk for superficial fungal infections. For example, in a case series of consecutive patients diagnosed with tinea capitis in India, 90% of cases occurred in children younger than 15 years and 75% of cases among individuals who belonged to lower income socioeconomic groups.^86^ In Nigeria, among schoolchildren diagnosed with superficial fungal infections, infection was more likely to occur in children from families of lower socioeconomic status, with 66% of cases reported, whereas those from higher socioeconomic status were overrepresented (57%) in the control group.^87^ In the U.S., lower socioeconomic status has been shown to be associated with increased risk of onychomycosis.^88^

### Housing, basic amenities, and the environment

The residential environment, encompassing both housing conditions and community characteristics, exerts a substantial influence on individuals well-being and health outcomes. In most settings the physical built environment has a significant impact on public health and health outcomes, as it influences factors such as physical activity levels, access to healthy food, good quality indoor air, and water, social interactions, medical services, and overall safety and well-being.

Currently, three billion people—40% of the global population—lack facilities at home to wash their hands with soap and water, and nearly half of schools and 43% of healthcare settings lack these amenities.^9^ In addition, over 90% of people globally breathe outdoor air with pollution levels exceeding WHO air quality guideline values, which contributes to over 7 million deaths annually.^9^ Even within industrialized Western countries, the residential environment is a major determinant of health outcomes. In western countries, for instance, high levels of crime and violence, high concentrations of convenience food outlets, lack of grocery stores, limited access to fresh, affordable, and nutritious food, and high concentrations of liquor stores and tobacco products correlate strongly with poor physical and mental health outcomes.^89^, ^90^, ^91^, ^92^ Higher social vulnerability has also been shown to be associated with unsafe housing environments.^93^

There is a relationship between population density and risk for developing an IFI. Rates of histoplasmosis tend to be higher in individuals who live in rural lower income areas, as previously discussed. There is likely an interplay between living in rural areas, lower income, and environmental as well as occupational exposures to fungi.^85^^,^^94^ Conversely, some IFIs are diagnosed more frequently in urban areas, including aspergillosis, coccidioidomycosis, and pneumocystosis.^85^^,^^95^ Aspergillosis and pneumocystosis may be diagnosed more frequently in urban areas due to the risk factors for this infection, such as hematologic malignancy, immunosuppressive conditions, transplantation, and closer proximity to tertiary medical centers treating patients with these conditions. Outbreaks of IFIs, particularly aspergillosis, have been associated with construction work in the vicinity of hospitals, which can aerosolize conidia,^96^ further contributing to the risk for IFIs from aerosolized conidia in urban areas.

Globally, higher rates of dermatophytoses have been observed in areas with higher population density or overcrowded areas such as in homes and schools. In Iran, higher rates of tinea capitis were associated with larger families and larger class size in schools.^97^ Similarly, among school-age children, sharing a bed with more than three people was associated with higher rates of superficial fungal infections of the skin in southern Tanzania.^98^ Poor living conditions were associated with higher rates of superficial fungal infections in South Western Nigeria,^87^ and in another study in Nigeria, diagnosis of tinea capitis occurred more frequently in areas of frequent interaction with soil and animals.^99^

In India, which represents the country with the highest reported rate of mucormycosis, studies have shown that some individuals acquired COVID-19-associated mucormycosis (CAM) at home in their bedrooms, where higher Mucorales spore counts were measured in the rooms of individuals convalescing from COVID-19 infection compared to homes of individuals not affected by CAM.^100^^,^^101^

In addition to invasive infection, outdoor and indoor air pollution in residential environments, including high concentrations of fungal pathogens, has a significant adverse effect on human health by enhancing the production of allergens.^102^ *Stachybotrys chartarum* growth can occur on water-damaged areas of buildings and has been associated with acute idiopathic pulmonary hemorrhage in infants, although this association is controversial.^103^ Some fungal propagules contain pro-inflammatory and pro-allergenic compounds which can cause allergic response. For example, *Aspergillus fumigatus* contains eighty allergic proteins capable of inducing IgE responses, *Alternaria* spp. contains twelve, and *Cladosporium herbarum* eight allergens.^104^ It is estimated that 6% of the general population and 20–30% of allergy-predisposed individuals are allergic to environmental fungi.^105^ In addition, an estimated eight to ten percent of individuals with cystic fibrosis experience allergic bronchopulmonary aspergillosis.^104^ Lastly, *Exophiala dermatitidis* colonizes the respiratory tract of up to nineteen percent of individuals with cystic fibrosis^106^^,^^107^ and can cause infections as well.^108^

Besides the residential environment, recreational activities can contribute to fungal exposure and infection. For instance, outbreaks of blastomycosis infections have been associated with environmental exposure during recreational activities. One study in Wisconsin, U.S. documented a high incidence of blastomycosis infection associated with visitation to rivers or associated waterways, suspected to reflect recreational exposure.^109^ Another cluster was documented among two school groups who visited an environmental camp in northern Wisconsin, with soil at a beaver pond near the camp thought to be the cause.^110^ Lastly, overcrowded housing may have contributed to a large outbreak of blastomycosis in Wisconsin clustered among households which primarily affected people with Hmong ethnicity, for whom larger average household sizes have been documented.^111^ In addition, an outbreak of *Cryptococcus gattii* occurred on Vancouver Island in British Columbia, Canada. *C. gattii* was cultured from a number of environmental samples, with the highest concentration occurring at a park where almost half of those involved in the outbreak had visited within the prior 12 months.^112^

### Access to affordable and high-quality health services

A lack of timely access to affordable health services is associated with worse health outcomes. Living far from a health care center, fear of deportation if living in a county without documentation, high medical costs, or lack of understanding of how to access health care are among the more common reasons why individuals may lack access to high-quality health care. In the U.S., adults without health insurance are less likely to receive preventive services for chronic medical conditions that predispose to IFI such as poorly controlled diabetes mellitus, especially in the setting of widespread glucocorticoid use,^113^ cardiovascular disease, and cancer care.^114^ Conversely, health insurance is associated with improved access to health services and basic clinical services,^115^^,^^116^ increased rates of detection of diabetes mellitus, lower rates of depression, and reduced financial strain.^116^ Globally, approximately half of the population lacks access to health care, with an additional 100 million driven into extreme poverty by the cost of health care.^117^ Additionally, many communities globally affected by communicable diseases such as neglected tropical diseases (NTDs) are of low socioeconomic status and lack access to health services.^118^ Studies have shown that expanding health coverage decreases health disparities,^119^^,^^120^ and it is likely that health disparities are lower in settings with universal access to health care compared to settings that lack health care access for all.

Higher incidence of histoplasmosis has been associated with underlying medical conditions such as poorly controlled diabetes mellitus, autoimmune conditions such as rheumatoid arthritis, inflammatory bowel disease, psoriasis, and solid organ or hematopoietic stem cell transplant.^94^ Poorly controlled diabetes mellitus is a well-known risk factor for a number of IFIs, including mucormycosis.^85^^,^^121^ Severe COVID-19 infection, especially in the setting of widespread glucocorticoid use,^113^ has emerged as another major risk factor for mucormycosis^121^ and invasive aspergillosis,^12^ including a large mucormycosis outbreak during the COVID-19 delta wave in India.^121^^,^^122^ In one study, access to advanced COVID-19 treatment—including supplemental oxygen, remdesivir therapy, and ICU admission–was associated with lower CAM risk in resource-limited settings.^123^ There is also a clear association between prior pulmonary tuberculosis with cavitation and subsequent chronic pulmonary aspergillosis, which is a significant health problem in tuberculosis-endemic settings.^124^

Recently, an outbreak of fungal meningitis among patients who received procedures under epidural anesthesia at two clinics has been reported in Matamoros, Mexico. *Fusarium solani* species complex has been identified as the causative pathogen, although the exact source is still unclear.^125^ Many of these affected individuals were from the U.S. and involved in medical tourism–seeking lower-cost procedures with shorter waiting times. Increased risk for candidemia has been associated with poor health care infrastructure and access in lower income countries.^126^ Overcrowded intensive care units, insufficient air conditioning/ventilation systems, and lack of efficient infection control services predispose individuals from lower income countries to a greater risk for hospital and ICU outbreaks of IFIs, as recently shown for COVID-19-associated pulmonary aspergillosis, CAM, and CNS fusariosis outbreaks in Mexico,^123^^,^^127^^,^^128^ as well as invasive *Candida* infections caused by fluconazole resistant *C. parapsilosis*^129^ and *C. auris*.^130^ Cryptococcosis is associated with advanced HIV infection as well as other medical comorbidities such as diabetes mellitus, corticosteroid use, and malignancy.^131^^,^^132^ Improved primary prevention, affordable access to early diagnosis, and treatment of chronic medical conditions would likely reduce the risk for IFIs.

### Substance use, crime, and incarceration

Substance use has been associated with a number of IFIs. Injection drug use (IDU) is a common cause of bacterial and, less frequently, fungal endocarditis. The latter is mainly caused by *Candida* spp., followed by *Aspergillus* spp. and rarely *Histoplasma* spp. or other endemic fungi,^133^ and mucormycosis.^134^, ^135^, ^136^ Cigarette smoking has been associated with an increased risk for coccidioidomycosis,^137^^,^^138^ paracoccidioidomycosis,^139^ and cryptococcosis^57^. Smoking marijuana has been linked to invasive pulmonary aspergillosis, possibly due to *Aspergillus*-contaminated leaves from the *Cannabis sativa* plant.^140^, ^141^, ^142^ In one study, persons who use cannabis were 3.5 times more likely to develop an IFI compared to non-cannabis users.^141^ Electronic cigarette use or vaping has been associated with invasive pulmonary aspergillosis infection^143^ and *Candida albicans* growth in the gingiva of the oral cavity.^144^

Multiple studies have shown increased risk for coccidioidomycosis infection for incarcerated individuals in areas endemic for *Coccidioides* spp., such as in the Central Valley in California^145^, ^146^, ^147^ as well as state prisons in other regions in California.^148^ Those who are incarcerated are more likely to be men and BIPOC individuals as Black or African American men between ages 20 to 34 have the highest rate of incarceration in the U.S.^149^ In addition, compared to the general population, incarcerated individuals are more likely to have high blood pressure, asthma, cancer, arthritis, and infectious diseases such as tuberculosis, HIV, and hepatitis C infection.^150^, ^151^, ^152^

### Structural conflict

Structural conflict such as armed conflict and/or the occupation by foreign armed forces can result in the displacement of populations, loss of income and shelter, the destruction of social networks and physical infrastructure, and violence and human rights abuses, exposing underlying inequities in SDOH. For instance, in Afghanistan, thousands of individuals were injured by unexploded ordinance associated with the ongoing war. One study showed that the vast majority were men and boys, with most injuries occurring in children playing and tending to animals, and adults engaged in economically necessary activities such as farming and traveling.^153^

In a study of combat casualties in Afghanistan during a 5-year period, it was estimated that IFIs accounted for up to 13% of associated morbidity and mortality. Risk factors included sustaining a dismounted blast injury, experiencing a traumatic transfemoral amputation, and requiring large-volume blood transfusions.^154^ Higher risk for IFIs has also been shown to be related to lower elevations, warmer temperatures, and greater isothermality, such as in southern Afghanistan where molds grew from 61% of wound cultures.^155^ Mucorales are the most frequent cause of fungal infections complicating combat-related injuries, followed by *Aspergillus* spp. and *Fusarium* spp.^156^^,^^157^ In addition, species not commonly seen in sinopulmonary mucormycosis are more common in theaters of war, such as *Apophysomyces* spp., *Saksenaea* spp., and *Lichtheimia* spp.^158^ Treatment of combat related IFIs can be particularly challenging, often requiring extensive debridement^159^ due to imperfect penetration of antifungals into wounds and often limited medical infrastructure in conflict zones.^160^ Lastly, the ongoing conflict in Sudan has led to the suspension of activities at the Mycetoma Research Center in Khartoum, the only institution in the world that specializes in the treatment of this disease. Thousands of patients now lack access to treatment.^161^

People displaced from structural conflict are also at an increased risk for fungal infections. For instance, superficial dermatophytoses were common among displaced Rohingya in a refugee camp in Bangladesh,^162^ refugees from camps in Sudan,^163^ Syrian refugees in a refugee camp in Jordan,^164^ and refugees settling in the U.S.^165^

### Food insecurity

Food insecurity is defined as a household-level economic or social condition of limited or uncertain access to adequate food.^166^ Globally, between 702 and 828 million people–approximately 10% of the global population–were affected by hunger in 2021. This number grew by 150 million since the start of the COVID-19 pandemic, with global conflict, climate extremes, economic instability, and growing inequality also contributing.^167^ In the U.S., 13.5 million households were food insecure at some point in 2021.

Food insecurity and malnutrition can be a predisposing factor for IFIs. As an example, *Pneumocystis jirovecii* infection has been associated with low body weight and protein deficiency in children.^168^

In addition, fungal plant pathogens pose a significant threat to food security and contribute to the US$220 billion estimated annual crop losses caused by pathogens and other pests.^169^ For instance, the plant fungus *Claviceps purpurea* can contaminate rye, wheat, and other cereals and cause ergot, which can manifest in a diverse array of symptoms in humans, including tremors, delusions, seizures, muscle spasms, and hallucinations. The plant pathogen *Phytophthora infestans* causes an estimated US$6 billion in potato losses and management costs annually.^170^ *Pyricularia oryzae* is a major rice pathogen, causing 10–35% of loss to harvests, and *Puccinia graminis* can cause up to 70% of wheat crop losses. Numerous *Fusarium* spp. are pathogenic to a diverse array of fruits and vegetables,^171^ causing up to 50% yield losses in fruit and vegetable crops such as bananas, pineapples, lentils, tomatoes, and peas.^172^ *Nosema* spp. have been implicated in colony collapse disorder and declining honeybee populations, which has had a significant impact on crops pollinated by the honeybee.^173^^,^^174^ Overall, fungi destroy a third of all food crops annually, resulting in significant economic loss and contributing to global poverty.^175^ A significant fungal outbreak involving one or more crops could have devastating consequences for the global food supply, which would most impact those from lower income settings. Lastly, although fungicides may help prevent crop loss, they can also lead to the emergence of antifungal resistance, as has been shown against all classes of antifungal drugs.^176^^,^^177^

## Interventions to reduce the risk for fungal infections

**Fig. 2:**
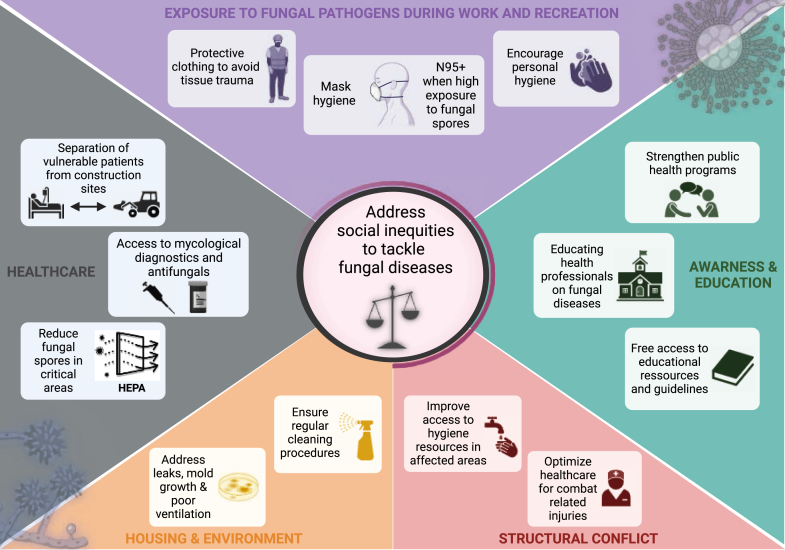
**Strategies to decrease risk of fungal infections by SDOH**.

Reducing the risk for fungal infections involves addressing underlying disparities in SDOH that increase the risk for acquiring these infections. Some of these interventions may be applied generally, while specific interventions may be needed to target individual risk factors. Social inequities, such as poverty, limited access to food and health care, inadequate housing, and educational disparities can contribute to an increased risk for developing fungal infections, and addressing these iniquities is multi-faceted (Fig. 2).

Susceptible individuals may develop IFIs after traumatic inoculation of fungal propagules in wounds. Thus, at-risk individuals should avoid tissue trauma when working in surroundings likely to have high concentrations of fungal propagules, such as in soil. PPE such as protective clothing and gloves can help to protect the skin from contact with mycoses that can potentially cause infection.^178^

In addition, addressing housing issues such as leaks, mold growth, or poor ventilation can also decrease the risk for acquiring a fungal infection, and this is particularly important for immunocompromised individuals. Mitigating actions to reduce the risk for developing fungal infections during construction work may help reduce outbreaks in hospitals. Such actions may include educating construction workers and hospital staff on preventative strategies to reduce dust production during construction activities, implementing physical barriers to minimize dust spread, and implementing filter systems to reduce fungal burden in the air. High-efficiency particular air (HEPA) filters, for example, have been proven to efficiently filter particles with a diameter of more than 0.3 μm^179^ and can prevent airborne IFIs in critical areas, such as stem cell transplantation units.^180^ The implementation and enforcement of well-funded laws and regulations are crucial to address the discussed issues. Governments, along with regulatory bodies, have a responsibility to ensure their implementation and that strong labor laws and protections exist to protect workers from fungal infections. Stakeholders, including professional associations, tenants, landlords and private sector industries such as housing and agriculture, should prioritize these concerns by conducting regular checkups and air quality testing to safeguard the well-being of residents and workers. Employers must uphold the highest occupational safety and health standards, minimizing hazards through the provision of necessary mitigation strategies. These actions are vital in creating healthier and safer living and working environments for individuals and communities.

Access to affordable and quality health care is critical for individuals with underlying conditions associated with increased susceptibility to IFIs. In addition, health care institutions should have access to appropriate fungal diagnostics and antifungal treatment. Unfortunately, these resources are not equitably distributed globally.^181^ For example, 90% of health care institutions in Europe but only 70% of health care institutions in the Asia/Pacific region reported access to echinocandins to treat IFIs such as candidiasis and aspergillosis.^182^^,^^183^ Access to antifungals for the treatment of implantation mycoses, such as eumycetoma, actinomycetoma, cutaneous sporotrichosis, and chromoblastomycosis, continues to be problematic in lower and middle-income countries globally.^184^ Inequities in antifungal drug agents and diagnostics can lead to suboptimal management of IFIs in resource limited settings.

Awareness and education on the signs and symptoms of fungal infections is important for both the general population as well as health care workers, to ensure timely and effective management of these infections. Several guidelines, such as those published on the management of cryptococcosis,^185^^,^^186^ mucormycosis,^187^ endemic mycoses,^188^ and rare mold infections^189^ offer guidance on the management of these infections, including in resource-limited settings. In addition, in the oncology setting recommendations on how to avoid excessive fungal exposure have been published.^190^ Still, there remains a lack of guidelines for neglected fungal tropical diseases such as mycetoma and chromoblastomycosis, and the guidelines that currently exist need to be widely available and freely accessible to healthcare workers around the globe. More research should be done on IFIs associated with occupational health hazards and combat injuries, including better tracking of these fungal infections so those who manage these injuries are better able to diagnose and offer appropriate antifungal treatment. Lastly, although a number of surveillance networks already exist to monitor existing communicable and non-communicable diseases, including for emerging pathogens,^191^, ^192^, ^193^ more widespread global surveillance of fungi is warranted to mitigate the impact of these pathogens on the health of those most at risk.

## Discussion

SDOH play a significant role in global health inequity and are associated with increased risk for developing superficial and IFIs, as discussed in this review (Fig. 1). This is particularly true for working conditions, where the risk for developing an infection from fungi is highly associated with certain types of work, including construction, farming, landscaping, and other agricultural work. Lower income is related with increased risk for certain IFIs globally and, along with more crowded housing, is associated with risk for superficial fungal infections globally. Lack of access to quality health care, and the underlying medical conditions associated with it, is associated with increased risk for IFIs. Structural and armed conflicts are associated with risk for IFIs following traumatic injury, and displacement of people fleeing conflict is associated with superficial and likely subcutaneous fungal infections. Finally, several fungal plant pathogens pose a significant threat to food security, and this threat is expected to intensify with the progression of global climate change^194^^,^^195^ and the subsequent weather phenomenon will likely increase the vulnerability of communities to natural disaster globally.^196^, ^197^, ^198^, ^199^, ^200^, ^201^ These global changes are expected to predominantly affect those who are already at an increased risk for fungal infections due to inequities in SDOH. Of note, the current published literature likely significantly underrepresents the association between SDOH and IFIs outside of the U.S., where the risks likely mirror those found within the U.S.

To decrease the morbidity and mortality associated with fungal infections, more research needs to be dedicated to implementing interventions that can help decrease the acquisition of fungal infections in those most at risk. The gaps in evidence need to be addressed and a more systematic approach to understanding and addressing the impact of SDOH on IFIs globally–with a specific focus on low-resource settings–needs to be implemented. Well-funded regulations that hold employers, landlords, and companies accountable could help address SDOH, if they are enforced. In addition, more emphasis needs to be placed on decreasing the health inequities between low-income and high-income countries and within higher-income countries such as the U.S.

## Outstanding questions

An important question is how to best provide surveillance of fungal infections globally, particularly in resource-limited settings where the prevalence of fungal infections is likely grossly underestimated. Another challenge is how to capture data on SDOH at a global level so the association between SDOH and fungal infections can be better understood. From a programmatic level, what role do agencies such as the WHO play in monitoring SDOH and fungal diseases and the association between the two?

## Contributors

JDJ and MH conceived and designed the study. JDJ, JP, and MH wrote the initial draft. RS, MO, and ME produced figures. SW, RS, DS, ME, CDR, HS, OAC, GRT, and DPK provided critical comments. All authors read and approved the final manuscript.

## Declaration of interests

Conflict of Interest and Sources of Funding: **JDJ** received research funding from Astellas, F2G, and Pfizer—all outside of the submitted work. **JP** has received speakers’ fees from Gilead Sciences, Pfizer, Swedish Orphan Biovitrum, Associated of Cape Cod, served at advisor boards for Gilead Sciences and Pfizer and holds stocks of Novo Nordisk and AbbVie Inc—all outside of the submitted work. **RS** received speaker fees and travel support from Pfizer—all outside of the submitted work. **DS** received speaker fees from Pfizer—all outside of the submitted work. **OAC** reports grants or contracts from BMBF, Cidara, EU-DG RTD (101 037 867), F2G, Gilead, MedPace, MSD, Mundipharma, Octapharma, Pfizer, Scynexis; Consulting fees from Abbvie, AiCuris, Biocon, Cidara, Gilead, IQVIA, Janssen, Matinas, MedPace, Menarini, Moderna, Molecular Partners, MSG-ERC, Noxxon, Octapharma, Pfizer, PSI, Scynexis, Seres; Honoraria for lectures from Abbott, Abbvie, Al-Jazeera Pharmaceuticals/Hikma, Gilead, Grupo Biotoscana/United Medical/Knight, ISHAM Working Group, MedScape, MedUpdate, Merck/MSD,Noscendo, Pfizer, Shionogi, streamedup!; Payment for expert testimony from Cidara; Participation on a Data Safety Monitoring Board or Advisory Board from Boston Strategic Partners, Cidara, IQVIA, Janssen, MedPace, PSI, Pulmocide, Shionogi, The Prime Meridian Group; A patent at the German Patent and Trade Mark Office (DE 10 2021 113 007.7); Stocks from CoRe Consulting, EasyRadiology; Other interests from Wiley. **GRT** received research and consulting fees from Astellas, Cidara, F2G, Mayne, Melinta, Mundipharma, and served on the DRC for Pfizer—all outside of the submitted work. **DPK** received honoraria and research support from Gilead Sciences, Merck, United Medical, and Astellas Pharma. He received consultant fees from Astellas Pharma, Amplyx Pharmaceuticals, Ciadara Therapeutics, Mayne Pharma, and is a member of the Data Review Committee of Cidara Therapeutics, AbbVie, Scynexis, and the Mycoses Study Group–all outside of the submitted work. **MH** received research funding from Gilead, Astellas, Euroimmune, MSD, IMMY, Mundipharma, Scynexis, and Pfizer—all outside of the submitted work. All authors declare no conflict of interest.
